# Movement Diversity and Complexity Increase as Arm Impairment Decreases After Stroke: Quality of Movement Experience as a Possible Target for Wearable Feedback

**DOI:** 10.1109/TNSRE.2024.3439669

**Published:** 2024-08-20

**Authors:** Shusuke Okita, Diogo Schwerz de Lucena, David J. Reinkensmeyer

**Affiliations:** Max Näder Center for Rehabilitation Technologies and Outcomes Research, Center for Bionic Medicine, Shirley Ryan AbilityLab, Chicago, IL 60611 USA, and also with the Department of Physical Medicine and Rehabilitation, Northwestern University, Chicago, IL 60611 USA; AE Studio, Venice, CA 90291 USA.; Department of Mechanical and Aerospace Engineering, University of California Irvine, Irvine, CA 92697 USA, and also with the Department of Anatomy and Neurobiology, University of California Irvine, Irvine, CA 92697 USA.

**Keywords:** Wearable sensing, movement rehabilitation, activities of daily living (ADL), the inertial measurement unit (IMU), sample entropy, Fugl-Meyer upper exremity (UEFM) score, upper extremity (UE)

## Abstract

Upper extremity (UE) impairment is common after stroke resulting in reduced arm use in daily life. A few studies have examined the use of wearable feedback of the quantity of arm movement to promote recovery, but with limited success. We posit that it may be more effective to encourage an increase in beneficial patterns of movement practice – i.e. the overall quality of the movement experience – rather than simply the overall amount of movement. As a first step toward testing this idea, here we sought to identify statistical features of the distributions of daily arm movements that become more prominent as arm impairment decreases, based on data obtained from a wrist IMU worn by 22 chronic stroke participants during their day. We identified several measures that increased as UE Fugl-Meyer (UEFM) score increased: the fraction of movements achieved at a higher speed, forearm postural diversity (quantified by kurtosis of the tilt-angle), and forearm postural complexity (quantified by sample entropy of tilt angle). Dividing participants into severe, moderate, and mild impairment groups, we found that forearm postural diversity and complexity were best able to distinguish the groups (Cohen’s D=1.1, and 0.99, respectively) and were also the best subset of predictors for UEFM score. Based on these findings coupled with theories of motor learning that emphasize the importance of variety and challenge in practice, we suggest that using these measures of diversity and complexity in wearable rehabilitation could provide a basis to test whether the quality of the daily movement experience is therapeutic.

## I. Introduction

STROKE is one of the most prevalent diseases across the world [1]. It was reported that 80 percent of people experience upper extremity (UE) motor impairments following stroke [2]. Due to these impairments, people rely more on their less-impaired UE for daily activities [3], although the activity level of both UEs decreases as the impairment of the paretic arm increases [4], [5]. This phenomenon relates to the concept of “learned non-use” [6] and may contribute to poor adherence to home exercise programs [7] and reduce gains from home rehabilitation [8].

Following on these concepts, Han et al. suggested that people following stroke can be categorized as either “users”, or “non-users” based on how much they use their impaired arm in daily activities [9]. “Users” are hypothesized to enter a “virtuous cycle”, in which they actively use their impaired arm, resulting in an improvement of arm function [8]. “Nonusers”, in contrast, are hypothesized to enter a “vicious cycle”, in which their arm non-use leads to reduced arm function [8].

Escaping this vicious cycle is challenging due to multiple factors. Firstly, people generally don’t begin to use their impaired arm voluntarily unless its function reaches at least half of its normal functional ability [10], [11], which aligns with the threshold hypothesis proposed by Lucena et al. [9]. Secondly, even when individuals show improvement on clinical functional tests, such as the Action Research Arm Test (ARAT), this progress doesn’t necessarily translate to increased arm use in daily life, indicating a “translation gap” between arm functionality and actual use [12]. For example, a recent study found that a majority of stroke patients improved the capacity for UE activity as they recovered; however, they did not improve the actual performance of UE activity in daily life, as measured with a wearable sensor [13]. Further, patients typically spend limited time in therapeutic activities compared to non-therapeutic activities, with stroke patients spending only about 28% of their available time on therapeutic activities even in rehabilitation centers [14]. Finally, patients tend to overestimate the amount of their activity in home exercise programs [15].

We recently studied the potential of wearable feedback to help address several of these issues. By “wearable feedback”, we mean use of a body-worn sensor that provides feedback to modify behavior, such as a visually displayed number (e.g. # of steps) or vibration (e.g. when a goal is met). We developed the Manumeter, a wrist-worn magnetic array that senses the movement of a magnetic ring worn on a finger, indicating the number of hand movements on a display [16], [17], [18], [10]. We used the Manumeter to test the hypothesis that real-time, daily feedback could promote hand use in daily activities [18]. We found that providing three weeks of such feedback promoted a small but a statistically significant increase in the amount of hand movements. Further, UE movement ability, measured by the UE Fugl-Meyer (UEFM) Test and the Box and Block Test (BBT), modestly increased. Other studies of wearable feedback have also found modest benefits to providing wearable feedback of amount of hand use [19], [20], leaving open the question of whether there is a way to build on these results and increase the benefit of wearable feedback.

Here, we reason that people with a stroke not only use their UE less, but also have an impoverished daily movement experience because they don’t use their UE for a variety of tasks. This idea of impoverishment can be considered to relate to the idea of “movement quality”, but is somewhat different than how movement quality has typically been conceived in wearable sensor research. For instance, previous studies have found that people after stroke exhibit decreased movement smoothness both during supervised movement in the clinic [21] and during daily activities [22]. Therefore, movement smoothness has been suggested as a measure of movement quality, or, alternately, measures such as increased movement speed and range of motion, or decreased curvature of reaching movements have been suggested [23]. However, these measures of movement quality focus on the quality of individual movements rather than the overall quality of the entire daily movement experience.

The idea of impoverishment we propose here relates to the idea that severely impaired stroke subjects appear “stuck” in stereotypical, abnormal synergistic movement patterns due to reduced capability of the corticospinal tract and/or the presence of spasticity [24]. In any motor learning context, if a person only ever practices the same thing without variation and increasing challenge, it is difficult to improve in skill. Thus, we postulate that it is important for non-users to practice a more diverse set of challenging movements frequently throughout the day to enter a virtuous cycle that improves movement ability. This idea also relates to the idea of encouraging motor exploration after stroke ([25], [26]).

But what should this diverse set of more challenging movements look like, and how might it be sensed by a wrist-worn inertial measurement unit (IMU)? This study sought to identify statistical features of the distributions of daily arm movements, quantifiable with a wrist-worn IMU, that become more prominent as arm impairment decreases. We reasoned that better-recovered persons move in ways that would be advantageous to identify, to provide targets for less well-recovered persons to try emulating. Therefore, we examined how several candidate measures of the quality of movement experience varied with different levels of recovery. We did this by analyzing IMU data obtained from 22 persons with a stroke wearing a wearable sensor on the wrist. Here, we focused on three categories of statistical quantification: 1) those relating to distributions of acceleration and angular velocity magnitudes of the forearm movement throughout the day; 2) those relating to the distribution of forearm postures experienced throughout the day; and 3) those related to the complexity of forearm movements performed throughout the day. While the Manumeter also provides information on hand use and grasping, we specifically desired to focus on measures that could be implemented with conventional wrist accelerometers to increase the potential for widespread adoption. By identifying movement features that are more prominent in better-recovered individuals, we aim to provide targets for rehabilitation and recovery assessment that can be easily measured with wrist-worn devices.

## II. Methods

### A. Wearable Sensor and Experimental Protocol

The Manumeter is a wrist-worn device consisting of a six degrees-of-freedom (DOF) IMU with an accelerometer and a gyroscope (LSM6DSL; STMicrosystems, Switzerland), four magnetometers on four corners of the device, and an OLED display [16], [17]. For this study, we analyzed sensor signals only from the IMU, since we desired the results to be applicable to current, commercial wrist activity tackers. These IMU signals had been sampled at 52.6 Hz in a previous pilot study of the effectiveness of hand count feedback versus conventional home exercise [10]. Twenty-two participants (see overview of participants in Table I) wore the Manumeter once before the three-week hand-count feedback intervention and then once after the intervention. 10 data sets were lost because of data acquisition problems [18], [27]. Thus, in total, 34 data sets were used for the study. All trials were conducted at the University of California, Irvine, with each participant giving informed consent in accordance with a protocol sanctioned by the relevant Institutional Review Board. The level of impairment of these participants was quantified using two common clinical measures. The Box and Blocks Test (BBT) requires participants to pick up small blocks from a box and transfer them over a divider, transferring as many blocks as possible in one minute [28]. The Upper Extremity Fugl Meyer (UEFM) test measures the ability of participants to perform 33 different test movements, rating each 0, 1, or 2 and summing the points to get a total possible score of 66 [29].

### B. Processing of the IMU Data

**Fig. 1.**
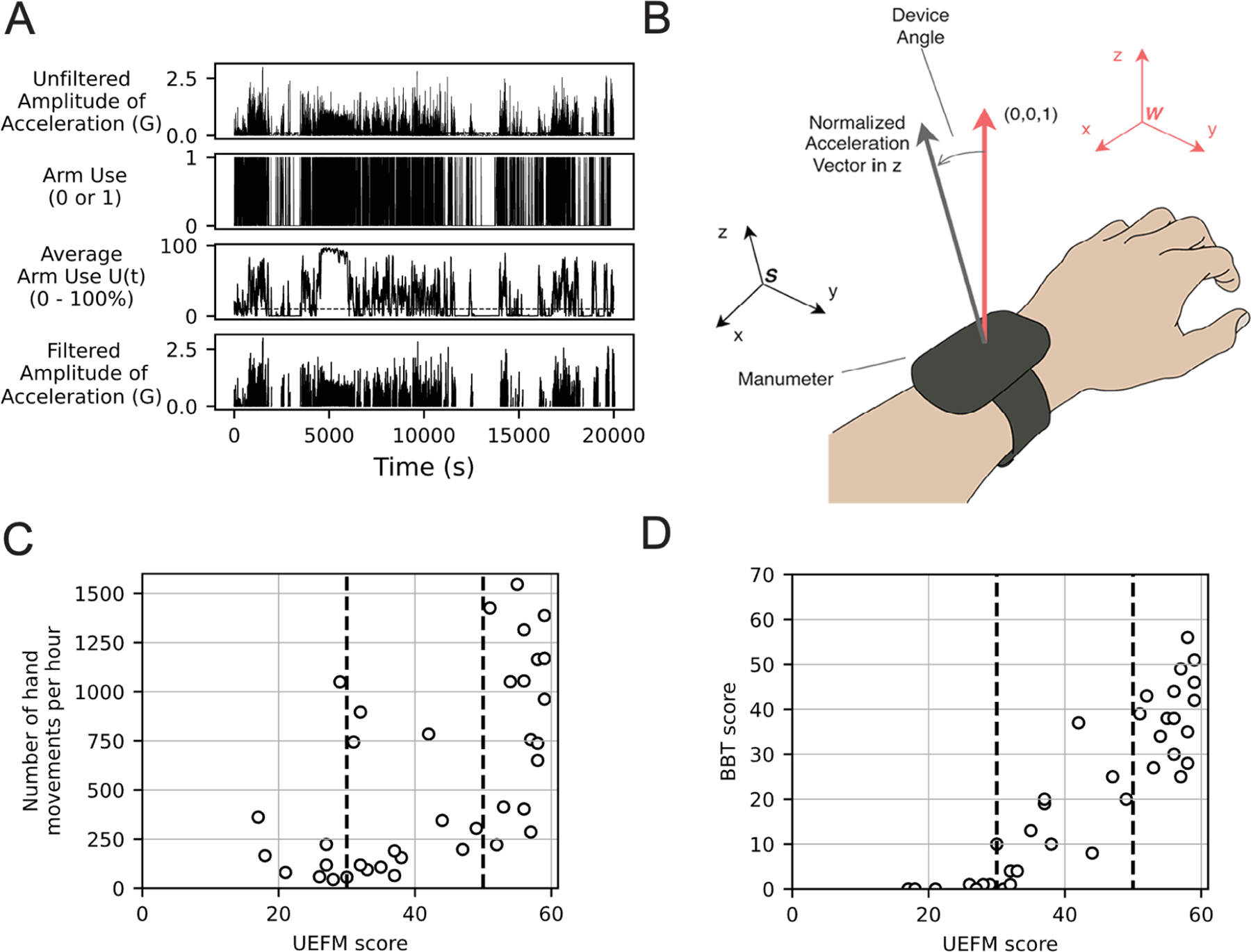
(A) Example of signals for one participant at different stages of preprocessing. From top to bottom: Raw amplitude of acceleration; Periods of active arm movement identified by the first threshold; Moving averages of filtered movements with a window size of 1000; Filtered acceleration using the second threshold. (B) The derivation of the device orientation with respect to acceleration vectors. The algorithm compares the tilt angle Θ between a vector in the world coordinate frame W and a vector in the sensor coordinate frame S. (C) The relationship between BBT Score and UEFM Score, replotted from [46]. Hand function emerges around UEFM = 30 (first dashed line) (D) The relationship between hand use intensity and the UEFM score (replotted from [10]). The hand use intensity is the number of hand counts detected from the Manumeter. Daily hand use emerges around UEFM = 50.

We used the Madgwick filter to subtract the gravity components from acceleration [30]. To identify the statistical properties of the participant’s arm activity, we needed to filter out periods of arm inactivity, which was done in the following way (Figure 1A). First, we removed any time periods when sensor values remained constant for over three minutes (cf. [31], [32]). Second, we introduced two measures of arm activity: (1) the instantaneous upper limb use score $u\left(t\right)$, and (2) the mean arm use score $u\left(t\right)$. $u\left(t\right)$ is a binary number where 1 represents that the magnitude of the arm acceleration is over a small threshold, chosen as described below.


(1) $$u_{i}\left(t\right)≜\left\{\begin{array}{l} 0\;(\text{UL is not in use at time }t)\\ 1\;(\text{UL is in use at time }t) \end{array}\right.$$

*$U\left(t\right)$* is the average of $u\left(t\right)$ over a sliding window with D=10 seconds long:

(2) $$U_{i}\left(t;D\right)≜\frac{1}{D}\;\int_{t-D}^{t}u_{i}\left(x\right)dx,t\in \left[D,T\right]$$

We identified a threshold for $U\left(t\right)$ such that if $U\left(t\right)$ were below that threshold, we considered the arm to be inactive at that movement and removed that data from our analysis. We will use the terms “total inactive time” for the total amount of time that we removed, and “total active time” for the time periods we kept. To choose thresholds for $u\left(t\right)$ and $U\left(t\right)$, we conducted a grid search in the range of [0.05, 0.30] for the threshold of $u\left(t\right)$, and [0.05, 0.30] for the threshold of $U\left(t\right)$, checking a combination of parameters that achieved a statistically significant correlation (see Supplementary Material). Our goal was to make the thresholds as lenient as possible to retain as much data as possible for analysis. However, we imposed the constraint that the chosen thresholds should produce a total inactive time that was as strongly correlated as possible with the impairment level measured by UEFM score. As a result, we chose a threshold of $u\left(t\right)=0.1\text{ G}$ and a threshold of $U\left(t\right)=10\%$ as parameters for the rest of the analysis.

### C. Forearm Orientation

Leuenenberger et al. previously used an estimate of the orientation of an IMU worn on the wrist to quantify functionally relevant arm movement of stroke patients, proposing it as a measure of movement quality [33]. Motivated by their approach and the principles in [34] and [35], we similarly estimated device orientation using the law of cosines [34]:

(3) $$\text{Θ}=\arccos\left(\frac{-a_{z}}{\sqrt{a_{x}^{2}+a_{y}^{2}+a_{z}^{2}}}\right)$$

where $a_{x}$, $a_{y}$, and $a_{z}$ represent the components of the measured acceleration vector along the x, y, and z axes, respectively, with respect to a sensor coordinate frame S (Figure 1). When a person’s hand is pronated (palm down) and their arm is positioned horizontally, the accelerometer/gyroscope sensor coordinates align with the indicated sensor frame. The positive X, Y, and Z axes correspond to the posterior, medial, and vertical directions, respectively. Angular velocity about the X axis (roll) represents forearm rotation, with supination and pronation indicated by positive and negative values. The Y axis (pitch) represents forearm flexion and extension, while the Z axis (yaw) represents wrist adduction and abduction. Equation 3 computes the angle Θ between the projection of the normalized acceleration vector in the sensor coordinate frame S, and a normal vector (0,0,1) with respect to a world coordinate W.

### D. Distributions of Acceleration and Angular Velocity Magnitudes, and Forearm Posture

To compare the shape of the statistical distribution of forearm posture, we used three standard statistical measures: kurtosis, skewness, and variance. Kurtosis relates to the sharpness of the distribution; the distribution becomes more rounded as kurtosis increases [55]. In our study, we computed excess kurtosis to describe the shape of the distribution of device angles. If the value of excess kurtosis is close to 0, the device angle distribution is close to a normal distribution. We also examined the distribution of acceleration and angular velocity measurements by dividing them into several bins. The range of acceleration was from 0–30 m/s^2^ and the range of angular velocity was from 0–100 deg/s. Bins were selected in 1 m/s^2^ increments for acceleration and 5 deg/s increments for angular velocity, resulting in 30 bins for acceleration and 20 bins for angular velocity (see further explanation in Appendix 6, in the supplementary material). The distributions were generated based on three separate groups.

### E. Sample Entropy

**Fig. 2.**
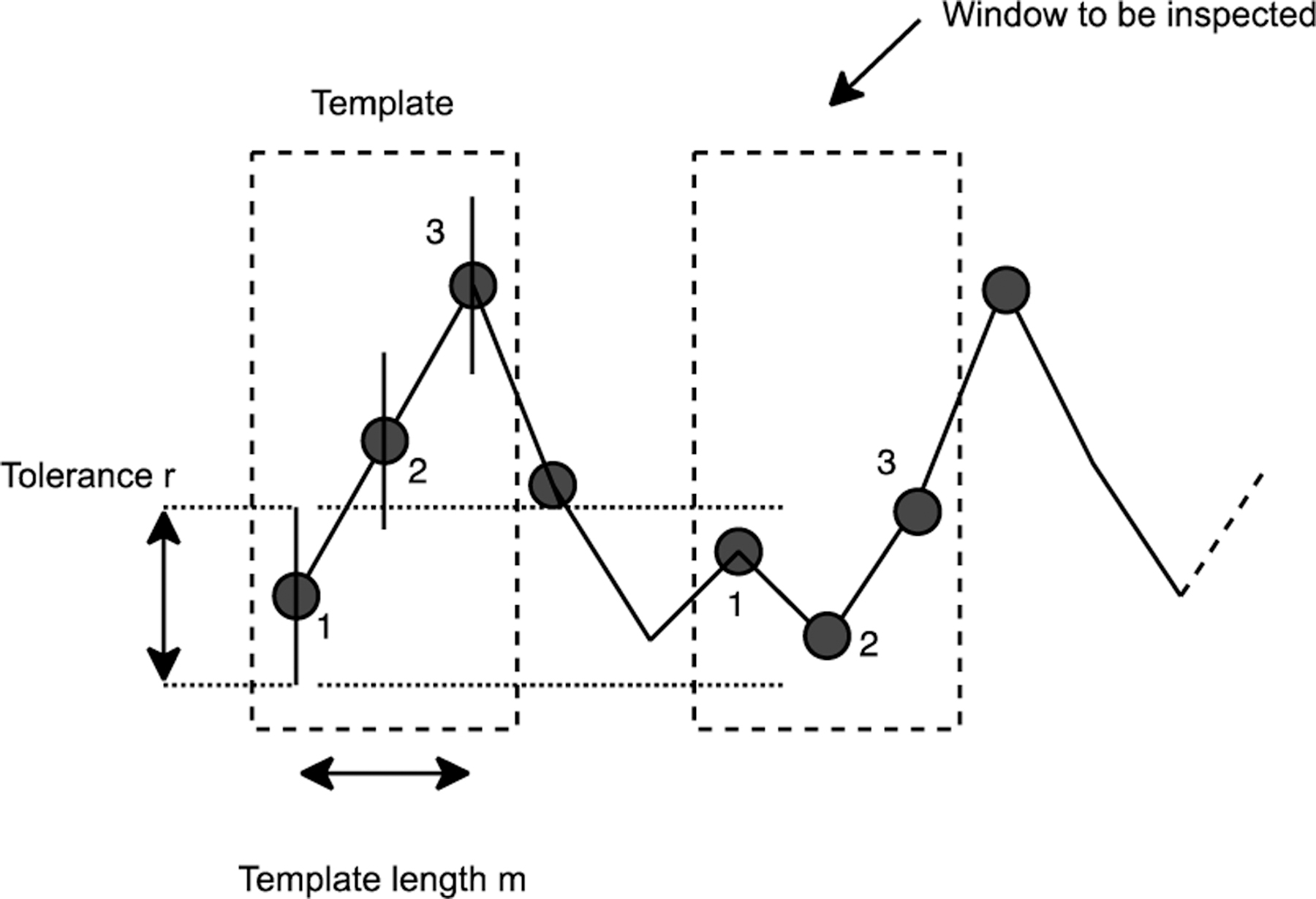
Overview of the Sample Entropy (SampEn) calculation. SampEn checks if there is a similar sequence in the sliding window compared to the template window.

To quantify the complexity of movement, we used Sample Entropy (SampEn), an established measure that quantifies the signal complexity of physiological measurements [36], [37], [38], such as EEG signals [39] and EMG signals [40]. SampEn is defined as follows:

(4) $$\mathrm{SampEn}(m,r)=-\ln\left[\frac{B^{m+1}(\gamma )}{B^{m}(\gamma )}\right]\;\;=-\ln\left[\frac{{\left(\text{N}-\text{m}-1\right)}^{-1}\sum_{\text{i}=1}^{\text{N}-\text{m}-1}{\text{B}}_{i}^{\text{m}+1}\left(\gamma \right)}{{\left(\text{N}-\text{m}\right)}^{-1}\sum_{\text{i}=1}^{\text{N}-m}{\text{B}}_{i}^{\text{m}}\left(\gamma \right)}\right]$$

where ${\text{B}}^{\text{m}+1}$ represents the number of matches of length m+1 with i+1 th template, and ${\text{B}}^{m}$ represents the number of matches of length m with i th template (Figure 2). We need to choose two key parameters to assess movements: (1) a template length m, and (2) a tolerance r. m represents a length of template to compare the signal to the rest of the data. SampEn computes the number of matchings with a template having a length m (i.e., $B^{m}\left(\gamma \right)$, and m+1 (i.e., $B^{m+1}\left(\gamma \right)$), and then computes the ratio of matching counts between $B^{m+1}\left(\gamma \right)$ and $B^{m+1}\left(\gamma \right)$. The tolerance r checks if a difference between a template and an inspected window is acceptable. In addition, we optimized the following additional parameters: (3) the segmentation length N, (4) the sampling rate, and (5) the type of sensor signals. The sampling rate is important because SampEn may not be good at identifying a complexity of high-resolution sensor measurements due to the nature of the algorithm [41]. To assess quality of movement with a smaller sampling rate than an original rate (i.e., 52.6 Hz), we down sampled IMU signals from 52.6 Hz to 26, and 13 Hz, respectively. We applied SampEn to (i) the amplitude of acceleration, (ii) the amplitude of angular velocity, and (iii) the tilt angle computed by Equation 3. We calculated the magnitude as an instantaneous measurement for each data point.

To decide the best combination of parameters for (1)-(5) that maximized a correlation between the sample entropy and UEFM scores, we chose parameters for each variable we studied (see Figure 1-3 in Supplementary Material). For acceleration, the template length $\left(\text{m}\right)$ was 2, the tolerance $\left(\text{r}\right)$ was 0.25, the segmentation length $\left(\text{N}\right)$ was 250, and the sampling rate was 26 Hz. For angular velocity, the template length $\left(\text{m}\right)$ was 5, the tolerance $\left(\text{r}\right)$ was 0.4, the segmentation length $\left(\text{N}\right)$ was 750, and the sampling rate was 52.6 Hz. For the tilt angle, the template length $\left(\text{m}\right)$ was 3, the tolerance $\left(\text{r}\right)$ was 0.35, the segmentation length $\left(\text{N}\right)$ was 250, and the sampling rate was 52.6 Hz.

### F. Identifying Measure Dependence on Arm Impairment

We hypothesized that subjects have a different statistical pattern of daily movement depending on their level of arm impairment and analyzed this dependence in two ways. First, we examined whether putative statistical measures of arm movement varied with UEFM score using regression analysis. Second, we compared groups of subjects divided into three discrete levels of impairment. A previous study [42] used Rasch analysis to propose that subjects could be split into severe, moderate, and mild groups based on UEFM cutoff scores of 19 and 47 out of 66. Here, we used a variant of this three-group approach based on our own analysis of clinical and wearable sensing data that we previously acquired, which allowed us to assign a distinct meaning for each group. Group 1 (severely impaired) and Group 2 (moderately impaired) are distinguished by their inability or ability to use their hand. Figure 1C shows the relationship between UEFM score and BBT score, a measure of hand function. We can see that the group of subjects with UEFM score < 30 pts only scored 0–2 blocks on the BBT test, meaning they had little to no hand function. Groups 2 (moderately impaired) and 3 (mildly impaired) are distinguished by whether they use their hand in daily life. Figure 1D shows the relationship between hand use, measured with the Manumeter, and UEFM score, replotted from [18]. Subjects with UEFM score ≥ 50 points (approximately) show a greater amount of hand use in the real world. Thus, we used UEFM scores of 30 and 50 as thresholds to split subjects into three groups with distinct meanings, for whom we then compared the statistical properties of arm movement.

### G. Statistical Analysis of Movement Experience Quality Features

The left column of Table II provides a list of sensor variables (or “movement experience quality features”) that we created, based on distributions of acceleration or speed, parameters describing the distribution of forearm postures, and signal complexity measures. As one feature, we computed the ratio of magnitude of acceleration and the ratio of angular velocity (see Appendix 6 in supplementary material for methodology). We performed linear regression between each metric and the UEFM score. To compare statistical differences between the three groups assigned by impairment level, we used analysis of variance (ANOVA). In order to find a feature that distinguished one group from another, we conducted an unpaired t-test. To assess how much a group was different from another by a selected measure, we computed an effect size using Cohen’s D [43].

We also used a multiple linear regression model to predict UEFM score. To check the multi collinearity between candidate features, we computed the variance inflation factor VIF between the variables:

(5) $$VIF=\frac{1}{1-R_{i}^{2}}$$

where $R_{i}^{2}$ represents the coefficient of determination. We conducted variable selection using the backward elimination [44]. In the ordinary least square (OLS) regression, we removed the variable having the highest p-value one by one until the model had a single variable, where the p-value was obtained by testing if the variable was is different from zero using a t-test. Then, we chose the model having a minimum Akaike Information Criterion $\left(\text{AIC}\right)$, a measure assessing the quality of the model [45], [46]. AIC is often favored over R^2^ because it takes into account both the goodness of fit and the complexity of the model, helping to avoid overfitting and select a more parsimonious model that is likely to generalize better to new data [47], [48]. By minimizing AIC, moreover, we can select a model that provides a good balance between fitting the data well and having a lower complexity [49]. To compare the models, we computed differences between each AIC and the minimum AIC in backward elimination:

(6) $$\text{AlC}=2\text{k}-2\ln\left(\hat{L}\right)$$

(7) $$\text{Δ}=\text{AlC}-{\text{AIC}}_{\min}$$

where k represents a number of estimated parameters in the model, $\hat{L}$ represents the maximum value of the loglikelihood function of the model, and ${\text{AIC}}_{\min}$ denote the minimum AIC from a model. The minimum AIC is the value of AIC that corresponds to the best model among a set of candidate models. The difference between the minimum AIC and each AIC determines how acceptable it is to select a model; it is expected there is too much difference if AIC is larger than 2.0; Burnham and Anderson stated that there is a substantial empirical support if the delta of AIC is small enough (i.e., ∆ < 2.0) [50].

## III. Results

### A. Total Active Time

The 22 participants with a stroke donned the wrist-worn IMU after they left the laboratory following several hours of clinical evaluations (i.e. usually in the late morning or afternoon). The average duration of IMU recording for the rest of the day was about six hours. During this time, participants performed active movements for 1.8 hours on average, as detected by the activity filter described in the Methods section. Total inactive time computed with respect to wear time for the impaired arm decreased significantly from about 80% to 60% as a function of the UEFM score (See Figure 5 in Supplementary Material). Segmentation of active periods happened every 40 seconds on average across subjects, and the number of segmentations were 108 times per hour on average across subjects.

### B. Distributions of Acceleration and Angular Velocity Magnitudes

**Fig. 3.**
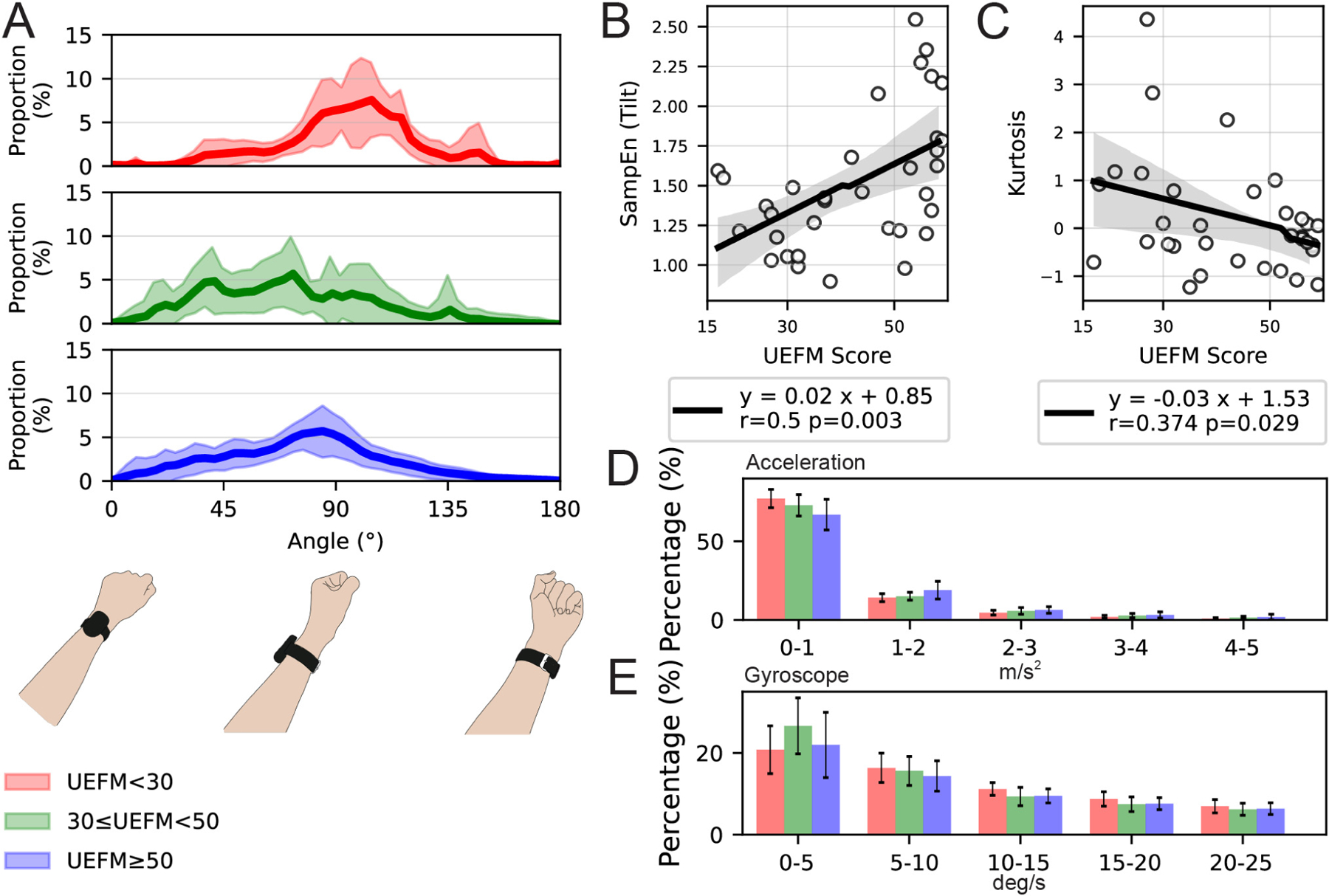
(A) The distribution of the device tilt angle and corresponding forearm postures with respect to the gravity, using 91 bins in range of 0–180 degrees (i.e., 2 degrees for one bin). The below three figures correspond to the device orientation. The red, green, and blue lines represent the mean of distribution for Group 1, Group 2, and Group 3, respectively. The shaded areas represent the confidence interval for each group. (B, C) The correlation between the UEFM score and: (B) SampEn of tilt angle, (C) kurtosis of tilt angle. (D, E) The proportion of participants’ movements as a function of magnitude of (D) acceleration magnitude and (E) angular velocity.

The distributions of the magnitude of acceleration and angular velocity for the groups of individuals in the three levels of impairment are shown in Figure 3D and E. Although the distributions were similar between groups, we observed differences between groups for the lowest acceleration range [0–1 m/s^2^], with the difference reversing in direction for the next highest acceleration range [1–2 m/s^2^]. To quantify this difference, we calculated a ratio of time spent at higher accelerations to time spent at lower accelerations. Specifically, we computed the ratio of the number of observations between 1–3 m/s^2^ divided by the number of observations between 0–1 m/s^2^). For velocity, we computed the ratio of observations between 5 deg/s divided by the number of observations between 5–15 deg/s.

Specifically, the more impaired subjects spent more time at low accelerations, and less time at higher accelerations. There was a statistically significant correlation between the acceleration ratio and UEFM score (Figure 6A in supplementary material), but not the angular velocity ratio (Figure 6B in supplementary material). Less impaired subjects displayed a higher acceleration ratio, meaning they spent relatively more time at a higher acceleration magnitude range. Mean of acceleration, and mean of angular velocity throughout the day also increased with FM score (Figure 6C and D, in supplementary material).

### C. Forearm Posture With Respect to Gravity

The distribution of forearm postures varied depending on group, with the severely impaired group showing a narrower distribution (Figure 3A). Kurtosis decreased significantly as a function of UEFM score (Figure 3B). Skewness also decreased as a function of UEFM score, but this decrease only approached significance (Figure 6E in supplementary material). Finally, variance increased significantly as a function of UEFM score (Figure 6F in supplementary material).

### D. Sample Entropy

We also tested a measure of movement complexity – the sample entropy – applied to the acceleration magnitude, the angular velocity magnitude, and the estimated forearm posture. Here, we calculated the acceleration magnitude as an instantaneous measurement for each data point. For each of these measures, movement complexity increased significantly as a function of UEFM score (Figure 3C, and Figure 6G, and H in supplementary material).

### E. Identifying the Measures That Most Effectively Distinguished Impairment Level

It is summarized in Table II which of the studied measures most effectively distinguished impairment level when subjects were grouped into the severe, moderate and mild impairment groups. Briefly, as a reminder, the group definitions were: Group 1: UEFM *<* 30, no hand function; Group 2: 30 ≤ UEFM *<* 50, hand function but low hand use; Group 3: UEFM ≥ 50, regular hand use. To distinguish Group 1 and Group 2, it was most useful to use kurtosis of the tilt angle distribution (Cohen’s D=1.1, t-test, p=0.028). To distinguish Group 2 and Group 3, it was most useful to use SampEn from tilt angle (Cohen’s D=0.99, t-test, p=0.017).

### F. Variable Selection for the Multivariate Model That Predicts the UEFM Score

We sought to develop a multivariate linear model that predicted UEFM score. We first removed variables that were not statistically significantly correlated with the UEFM score (Ratio of Angular Velocity, p=0.252; Mean of Angular Velocity, p=0.275). Then we conducted a backward elimination process with the remaining variables (as described in detail in the Methods) to understand which combination of features best distinguished groups, removing variables from the model one by one (see Supplementary Material). We observed the lowest AIC when we selected the number of variables to be two (Figure 7A in supplementary material). In this case, the selected variables were the kurtosis of the tilt distribution (i.e. forearm postural diversity) and the sample entropy of tilt (i.e., forearm postural complexity). We further examined the correlation coefficients between the variables (Figure 7B in supplementary material), seeking to identify if there was multicollinearity between any variables, which is a statistical phenomenon that occurs when two or more independent variables in a multiple regression model are highly correlated; a high correlation makes it difficult to determine the individual contribution of each variable to the dependent variable. To assess multicollinearity, we used the criterion that the Variance Inflation Factor VIF should be less than 5 [51]. The VIF measures the extent to which the variance of the estimated regression coefficients is increased due to multicollinearity. A VIF value less than 5 is considered acceptable, indicating that multicollinearity is not a significant concern. In our analysis, the correlation between Kurtosis and SampEn of tilt angle was weak (Pearson Correlation Coefficient = 0.22), and the VIF in Step 8 was 1.05 for both variables. These results suggest that multicollinearity is not a major issue for these variables in the model.

## IV. Discussions

The objective of this study was to identify statistical characteristics of daily arm movements that became more prominent as arm impairment decreased, based on data obtained from a wrist IMU worn by 22 chronic stroke participants during their day. Our focus here was on the statistical distributions of movement features across many arm movements made during the period the wearable sensor was worn. We hypothesize these features relate to the “quality of the movement experience” (which we will abbreviate QOME for this Discussion) over a period time. We identified several QOME-related measures that increased as UE Fugl-Meyer (UEFM) score increased: proportion of movements with higher forearm speed, forearm postural diversity (quantified by kurtosis of the tilt-angle), and forearm postural complexity (quantified by sample entropy of tilt angle). Dividing participants into severe, moderate, and mild impairment groups, we found that forearm postural diversity and complexity were best able to distinguish the groups. Specifically, to distinguish between severe and moderate impairment, kurtosis of the tilt angle, our measure of forearm postural diversity, was most effective. To distinguish between moderate and mild impairment, SampEn of the tilt angle, our measure of movement complexity, was most effective. The application of a multivariate modeling approach confirmed that these were also the best variables from among the ones we considered for predicting UEFM score. We discuss now these results as well as limitations and future directions.

### A. Quantifying Movement Diverisity Based on Forearm Posture (Tilt Angle) Distribution

We examined the simpler idea of tilt angle analysis with respect to gravity direction, first proposed by Leuenberger et al. [33] in the context of wearables for stroke rehabilitation, although our measure of tilt also took into forearm supination/pronation. They found that the impaired arm had lower tilt angle (or “elevation”) during performance of activities of daily living, found different probability distributions between the impaired arm and unimpaired arm, and suggested forearm posture as a potential target for improving patients’ upper arm dexterity. We found that tilt angle was distributed across a broad range of angles for participants with less impairment, but more narrowly concentrated for the most severely impaired group. It is an interesting question whether kurtosis is the best measure for our goal of quantifying movement diversity. We chose kurtosis because it is one of the primary methods to measure the “peakedness” of distributions, which fit well with what we intended. This paper can be viewed as a first attempt to confirm that kurtosis is indeed sensitive to stroke impairment. However, there may be even better measures of movement diversity, which future research should explore.

A narrow tilt-angle concentration is consistent with the concept of “postural stagnation” – i.e. that persons with severe impairment after stroke tend to keep their arm in a stereotypical posture when not using it. Previous studies have identified five different arm postures that stroke patients adopt, with two being most prevalent [52], [53]. In the present study, the greatest percentage of forearm postures of the most severe group was approximately 95-degree range, which may be due to the forearm remaining in the neutral position as part of one of these postures. Further, postural stagnation in severe impairment is consistent with the observation of stereotypical, abnormal synergistic movement patterns due to reduced capability of the corticospinal tract and/or the presence of spasticity [24]. A study of the ability of people with stroke to reach in a wide range of directions found that severely impaired individuals were constrained to reach in a narrow range [54].

Based on these results and the experimental findings, it seems probable that postural stagnation is a valuable concept for QOME metrics. As mentioned in the Introduction, if one only ever practices the same thing (i.e. holds the arm in a limited set of postures), it is difficult to improve in skill (i.e. learn to activate muscles for a wide range of arm postures and activities). Limitations of the tilt angle approach are that it cannot distinguish forearm supination/pronation from shoulder internal/external rotation and it doesn’t consider the posture of the whole body. Thus periods spent lying or reclining might introduce noise into the distribution. In activities of daily living, subjects sometimes change their posture, including lying down or reclining. These postural changes can alter the orientation of the device relative to gravity, which is critical when we are computing device angles based on raw acceleration data. As such, movements that do not involve significant limb activity but result in a change in device orientation can appear as anomalies or ‘noise’ in the distribution of our measured angles. Nevertheless, we found tilt-angle to be a valuable feature for distinguishing severely and moderately impaired participants.

### B. Quantifying Movement Complexity

To quantify movement complexity we focused on one of the many possible measure of signal complexity – entropy, a measure that has found application in human movement science for analyzing postural control, walking activity, spontaneous leg activity in infants, and finger force production (see review: [36]). Entropy features, specifically sample entropy, have been increasingly utilized in studies with wearable sensors, analyzing various biological signals to improve detection and prediction of motor impairments. In Parkinson’s disease research, Shawen et al. applied sample entropy to acceleration and angular velocity signals from skin-mounted sensors and smartwatches, enhancing tremor detection accuracy [55]. In autism studies, Konrad et al. used sample entropy to analyze wrist accelerometer acceleration signals, finding a moderate relation to motor coordination [56]. After stroke, sample entropy has been used to gain insight into UE muscle activity changes following robotic rehabilitation [57]. To our knowledge, this is the first report of showing the potential value of sample entropy of forearm posture, measured from a wrist worn IMU, for gaining insight into UE impairment after stroke. SampEn best distinguished participants with moderate and mild impairment, with the more mildly impaired group showing more complexity in their movements. This seems indicative that they typically achieved a richer daily movement experience, perhaps because they could use the arm in a wider variety of activities.

A limitation of the use of sample entropy is that it is strongly dependent on parameter selection [36]. To address this, we used a grid search to find the best combination of parameters (see Supplementary Material). Our finding that a template length of 4 was best is consistent with both recommendations and other studies in movement analysis [58].

Another potential limitation, particularly with a view toward implementation in a wearable sensor, is that SampEn can be computationally costly, with a complexity of $O\left(N^{2-\frac{1}{m+1}}\right)$, where N is the segmentation length and m is the template length [37]. Nevertheless, at least one study was able to implement SampEn in a wearable sensor for sleep research [59].

### C. Other Considerations: Movement Activity and Speed

Based on the inactivity filtering results, we found that participants with more severe impairment exhibited reduced UE activity. This finding is consistent with the theory of learned non-use [6] as well as previous studies using wearable sensing (e.g. [10]).

It was unexpected to us that movement diversity (quantified by kurtosis of tilt angle) and complexity (quantified by sample entropy) were the best features for distinguishing groups and predicting UEFM score, as opposed to movement speed. Nevertheless, distributions of movement speed did have some power to discriminate impairment level. In a study relevant for considering the potential of QOME wearable feedback, DeJong et al. examined the effect of instructing individuals with a stroke to move the UE more quickly as they reached and grasped a cup. They found that not only could the participants move their upper arm more quickly, but also that movement quality improved, as assessed by straighter reach paths and larger hand grip apertures [60]. Thus, by focusing on an easily instructed variable, other benefits related to movement quality could be obtained.

### D. Limitations and Future Work

Besides the specific limitations mentioned above with respect to the diversity and complexity measures, there are several other limitations to this work. First, we studied a relatively small list of potential measures related to QOME. Other measures are certainly possible and may be more strongly related to impairment level, or more valuable for encapsulating QOME. Second, the motivation for this work was to identify potential QOME measures that could be provided by a wrist-worn sensor to enhance rehabilitation. Third, we only used one technique to identify inactivity, which may limit the robustness and generalizability of our findings. Clearly, our hypothesis that providing feedback on QOME could be beneficial for rehabilitation requires testing in a clinical trial. We note that a large body of motor learning research supports the idea that challenging and variable task practice is beneficial [61]. Further, we have not identified whether and how people with a stroke can volitionally change a QOME measure. We aim to address these questions by embedding real-time QOME metrics in a wearable sensor and studying the effect of providing QOME feedback in future studies.

## V. Conflict of Interest

The author(s) declared the following potential conflicts of interest with respect to the research, authorship, and/or publication of this article: David J. Reinkensmeyer has a financial interest in Hocoma A. G. and Flint Rehabilitation Devices LLC, companies that develop and sell rehabilitation devices. The terms of these arrangements have been reviewed and approved by the University of California at Irvine, in accordance with its conflict-of-interest policies. All other authors declare that they have no competing interests.

## Supplementary Material

supp1-3439669

## Figures and Tables

**TABLE I:** Characteristics of the 22 Participants. SD = Standard Deviation

Age (Mean ± SD)	57 ± 15
Gender (Male[M])/Female([F])	16M / 4F
Months since stroke (Mean ± SD)	40 ± 33
Side of hemiparesis (Right [R]/Left [L])	12 R / 10 L
Type of stroke (Ischemic [I]/Hemorrhagic [H])	12 I / 10H
Box and Blocks Test (Mean ± SD for Number of blocks transferred in 60 seconds)	21 ± 18
Upper Extremity Fugl-Meyer (UEFM) Score (0–66, Mean ± SD)	40 ± 13

**TABLE II:** Summary of Movement Experience Quality Features Calculated Across the Recorded Movement Experiences

Kind	Sub kind	Correlation	R2	Effect size (p-value in t-test)				ANOVA (Group 1,2,3)	

				Group 1—Group 2	Group 2—Group 3	Group 1—Group 2,3	Group 1,2—Group 3	f	p-value
Tilt Angle	Kurtosis	0.383 (p=0.025)	0.15	1.145(0.028)	0.199(0.611)	1.529(0.054)	0.605(0.068)	6.40	0.005
	Skewness	0.314 (p=0.07)	0.10	0.292(0.548)	0.563(0.158)	0.616(0.156)	0.663(0.064)	2.05	0.146
	Variance	0.389 (p=0.023)	0.15	0.438(0.369)	0.395(0.348)	0.734(0.093)	0.592(0.096)	2.05	0.146

Ratio	Acceleration	0.435 (p=0.01)	0.19	0.561(0.255)	0.830(0.042)	0.791(0.072)	1.037(0.005)	4.70	0.016
	Angular Velocity	0.171 (p=0.333)	0.03	0.841(0.095)	0.445(0.261)	0.644(0.139)	0.079(0.821)	1.69	0.201

Mean	Acceleration	0.341 (p=0.049)	0.15	0.664(0.180)	0.553(0.166)	0.778(0.076)	0.772(0.032)	2.96	0.066
	Angular Velocity	0.188 (p=0.286)	0.04	0.116(0.810)	0.401(0.310)	0.340(0.429)	0.471(0.182)	0.93	0.407

SampEn	Acceleration	0.405 (p=0.017)	0.16	0.119(0.806)	0.805(0.048)	0.525(0.225)	0.862(0.018)	3.04	0.062
	Angular Velocity	0.41 (p=0.016)	0.17	0.610(0.217)	0.611(0.127)	0.868(0.049)	0.835(0.021)	3.58	0.04
	Tilt Angle	0.5 (p=0.003)	0.25	0.047(0.922)	0.993(0.017)	0.576(0.184)	1.106(0.006)	4.97	0.013
