# Supplementary material for: Movement Diversity and Complexity Increase as Arm Impairment Decreases After Stroke: Quality of Movement Experience as a Possible Target for Wearable Feedback
Source: IEEE Trans Neural Syst Rehabil Eng. Author manuscript; Available in PMC 2024 Oct 24. (PMC11500827; doi:10.1109/TNSRE.2024.3439669)
Supplement: supp1-3439669 [file NIHMS2017996-supplement-supp1-3439669.docx]

Supplementary Material

Appendix 1 - Optimization of Sample Entropy

The objective of this document is to describe results of optimization of sample entropy (SampEn) measures. This document describes a parameter study in sample entropy. The factors influences the computational complexity are (1) the template length m, (2) the segmentation length N, and (3) the sampling rate. the template length m changes the computational complexity because it requires more comparison with the remains of the data if we increase. (2) is especially important because we typically segment dataset and take an average of a value from each segment; this is because it’s computationally infeasible for us to apply SampEn to the entire data time series signals. Thus, it is common to take an average of SampEn with respect to a sliding window without overlapping. To find out the best parameters, we conducted the optimization using dataset from the Manumeter RCT.

We applied sample entropy to magnitude of acceleration, magnitude of gyroscope measurements, and tilt angle based on gravity direction (Figure 4, in the main text). For instance, younger patients have a better score in SampEn. We checked m=[2, 3, 4, 5] for template, and r = [0.1 - 0.3] for tolerance.

Figure 1-3 shows the parameter optimization for SampEn with acceleration, angular velocity, tilt angle, respectively. We made an ordinary least square fit for each combination and quantified an R2 squared value. As a result, we used different parameters for each variable (Table 1).


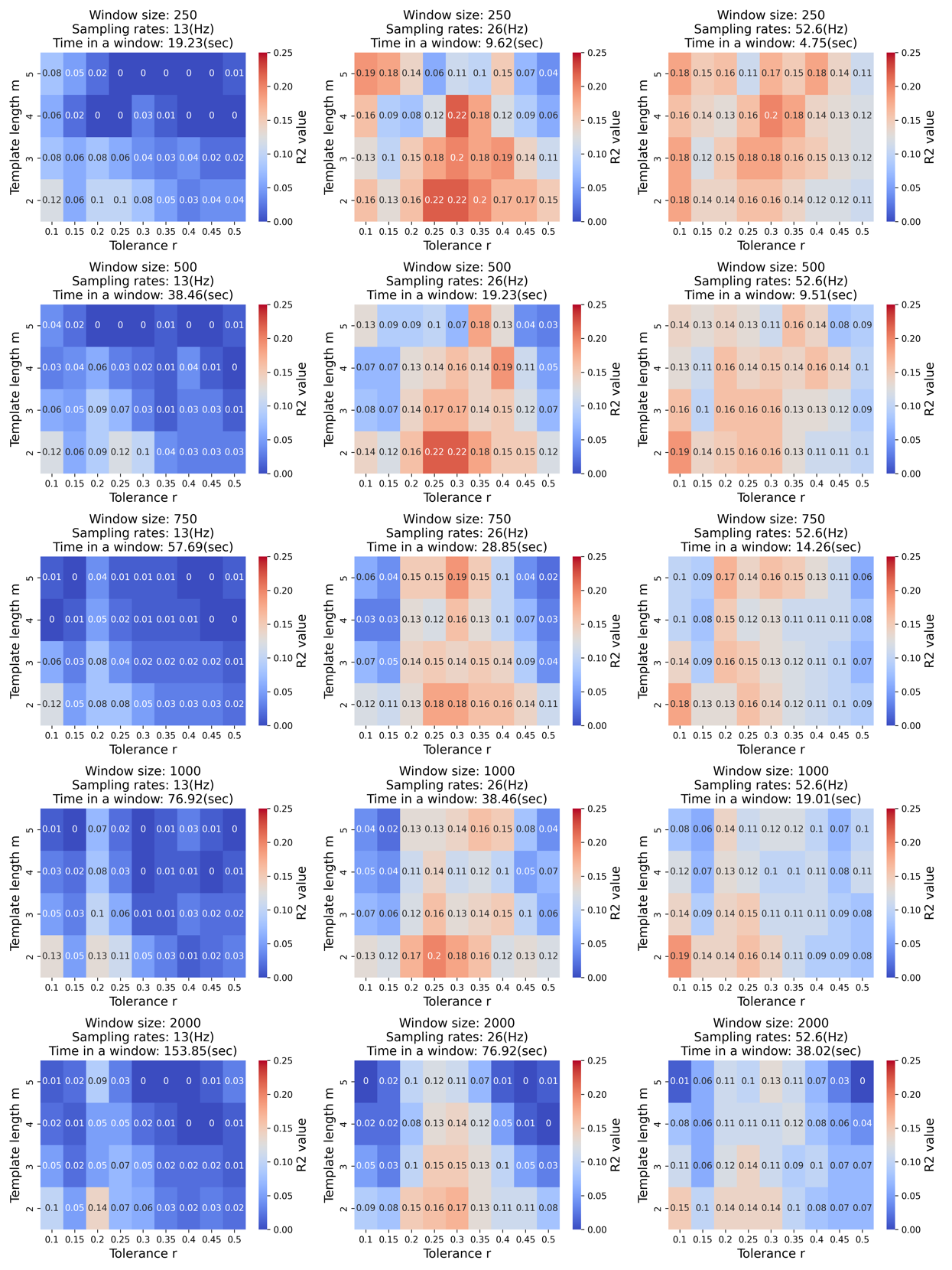


Figure 1. Parameter optimization of SampEn with acceleration.


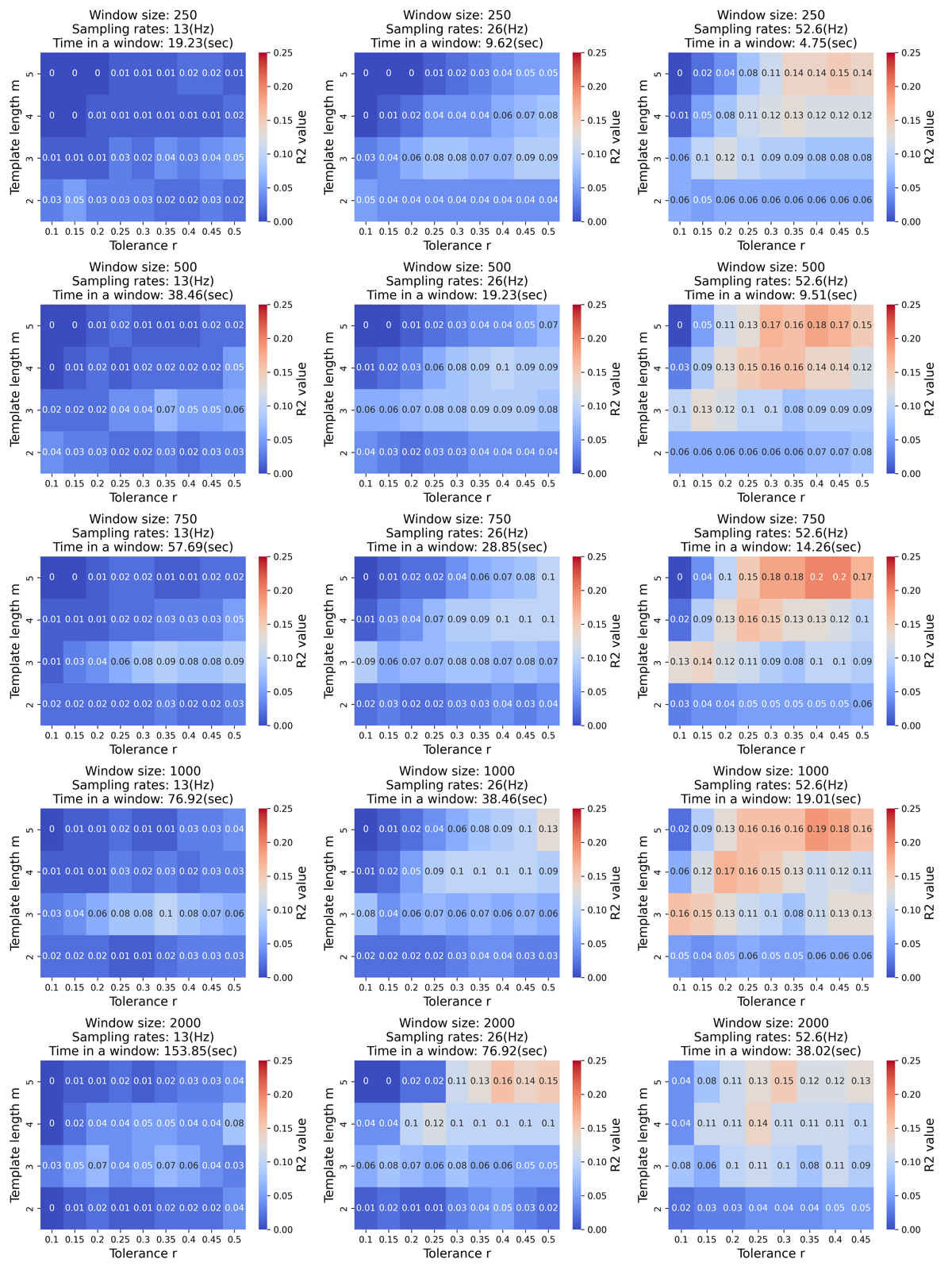


Figure 2. Parameter optimization of SampEn with angular velocity


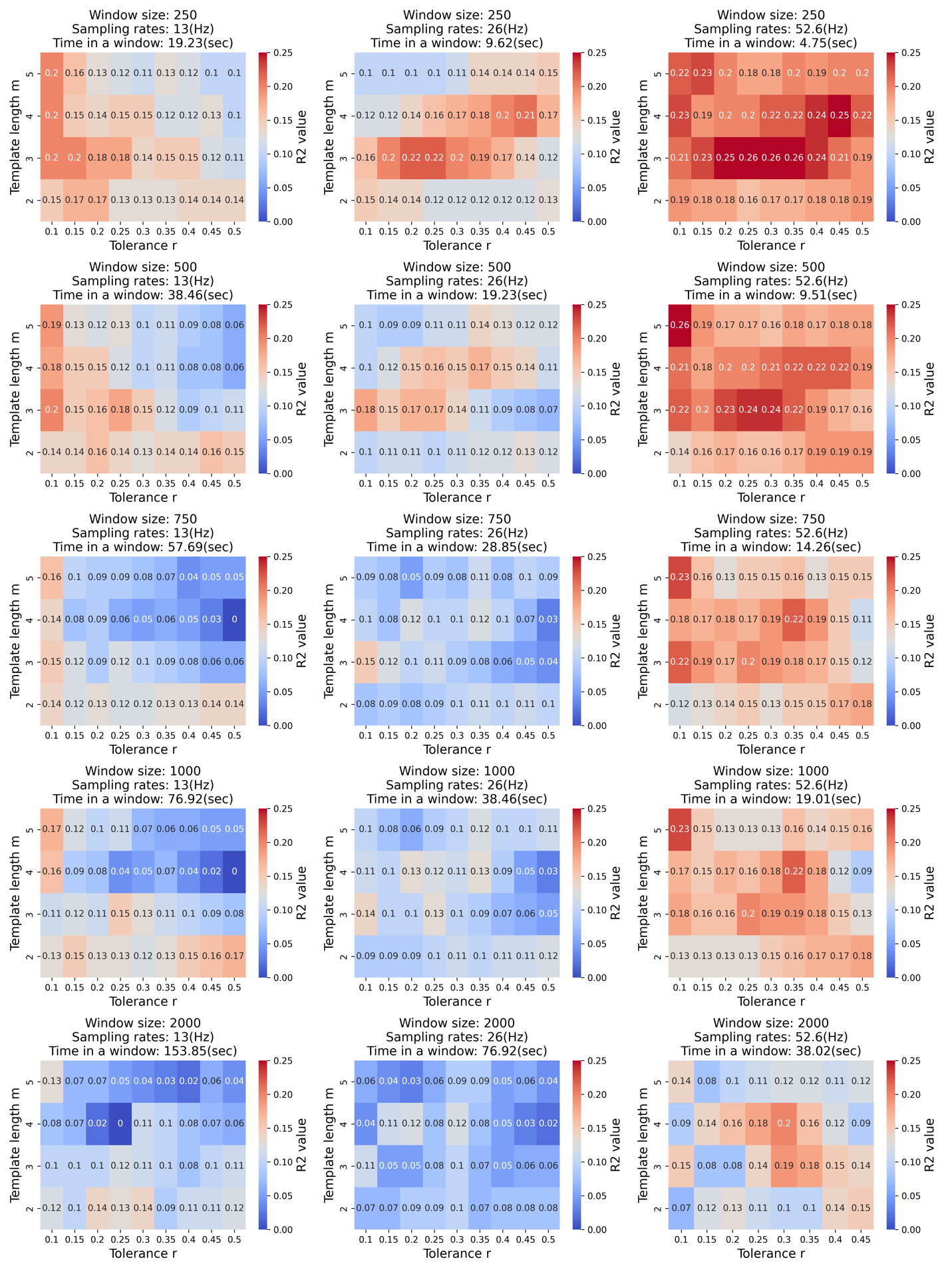


Figure 3. Parameter optimization of SampEn with tilt angle

Table 1. Parameter selection for three variables.

|  | Acceleration | Angular velocity | Tilt angle |
| --- | --- | --- | --- |
| Template length | 2 | 5 | 3 |
| Tolerance | 0.25 | 0.4 | 0.35 |
| Segmentation length | 250 | 750 | 250 |
| Sampling rate | 26 | 52.6 | 52.6 |

Appendix 2 - Optimization of inactivity filtering

This section describes the optimization of inactivity filtering. To find the optimal thresholds for inactivity filtering we conducted a grid search. We used the inactivity filtering using the methods in the main text. We chose the same parameter through the entire section to cut inactivity.


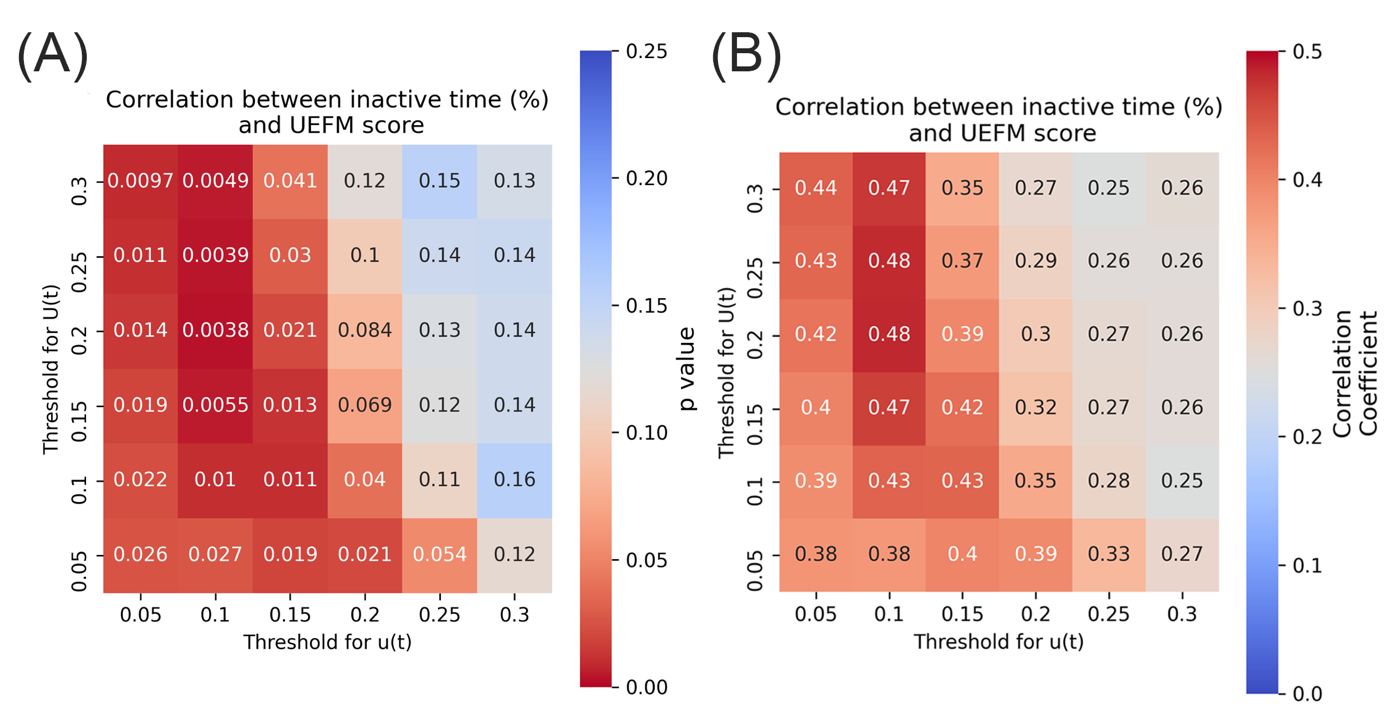


Figure 4. Optimization of thresholds.

Figure 4 shows the result of optimization. We can see a highly statistically significant difference from Threshold for u(t) = 0.1, and Threshold for U(t) = 0.1 (p=0.01). Since we would like to retain the information as much as possible, we chose this combination as the threshold for inactivity filtering.


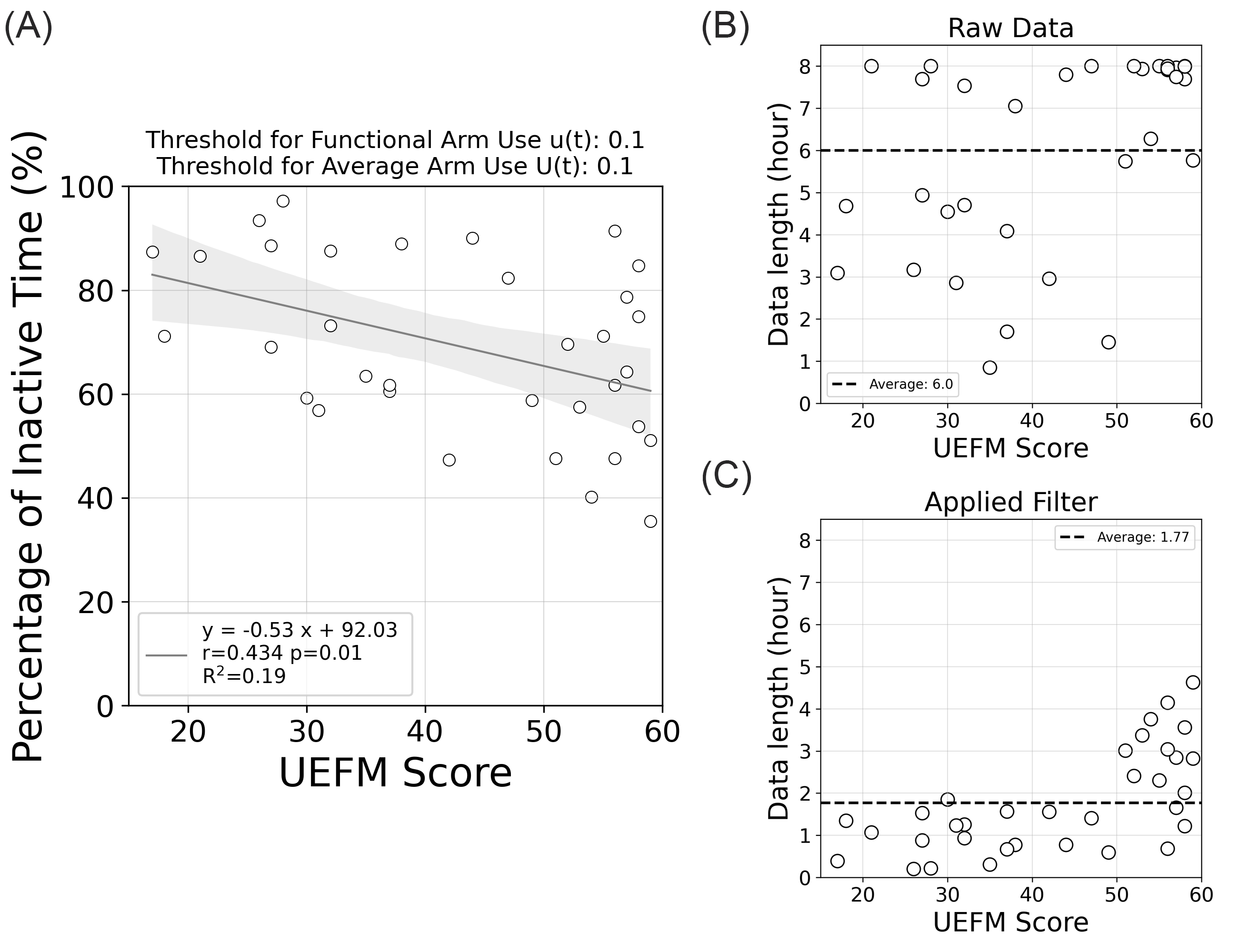


Figure 5. The best selection of percentage of the inactive time versus the UEFM score with chosen thresholds. (A) Percentage of the inactivity obtained from a black solid line represents a linear regression line (y=-0.53x + 92.03; Pearson correlation, r = 0.43, p=0.01, $R^{2}$=0.19) and the shaded area represents a confidence interval. (B) The amount of data and UEFM score without filtering. (C) The amount of data after filtering.

Appendix 4 – Variable selection with Backward Elimination

Table 3. Steps of the backward eliminations.

| Step | Variable | Coefficient | Standard Error | T | P-value | [0.025 | 0.975] | VIF |
| --- | --- | --- | --- | --- | --- | --- | --- | --- |
| 1 | Constant | 19.94 | 10.70 | 1.86 | 0.07 | -2.09 | 41.97 | 25.58 |
|  | Kurtosis of Tilt Angle | -2.01 | 2.76 | -0.73 | 0.47 | -7.69 | 3.67 | 2.29 |
|  | Skewness of Tilt Angle | -1.51 | 8.55 | -0.18 | 0.86 | -19.12 | 16.11 | 2.23 |
|  | Variance of Tilt Angle | 0.01 | 0.01 | 0.84 | 0.41 | -0.01 | 0.03 | 1.36 |
|  | Ratio of Acceleration | 21.82 | 24.25 | 0.90 | 0.38 | -28.12 | 71.76 | 2.35 |
|  | Mean of Acceleration | -9.85 | 12.31 | -0.80 | 0.43 | -35.21 | 15.50 | 3.17 |
|  | Sample Entropy (Acceleration) | -6.62 | 24.43 | -0.27 | 0.79 | -56.93 | 43.69 | 4.05 |
|  | Sample Entropy (Angular Velocity) | 1.75 | 2.23 | 0.79 | 0.44 | -2.84 | 6.34 | 2.38 |
|  | Sample Entropy (Tilt) | 12.15 | 10.42 | 1.17 | 0.26 | -9.31 | 33.62 | 4.29 |
| 2 | Constant | 19.23 | 9.73 | 1.98 | 0.06 | -0.77 | 39.24 | 21.98 |
|  | Kurtosis of Tilt Angle | -2.34 | 2.01 | -1.17 | 0.25 | -6.46 | 1.78 | 1.26 |
|  | Variance of Tilt Angle | 0.01 | 0.01 | 0.84 | 0.41 | -0.01 | 0.02 | 1.34 |
|  | Ratio of Acceleration | 21.87 | 23.79 | 0.92 | 0.37 | -27.03 | 70.77 | 2.35 |
|  | Mean of Acceleration | -9.50 | 11.92 | -0.80 | 0.43 | -34.00 | 15.00 | 3.09 |
|  | Sample Entropy (Acceleration) | -7.05 | 23.85 | -0.30 | 0.77 | -56.07 | 41.97 | 4.01 |
|  | Sample Entropy (Angular Velocity) | 1.83 | 2.15 | 0.85 | 0.40 | -2.59 | 6.24 | 2.30 |
|  | Sample Entropy (Tilt) | 12.11 | 10.22 | 1.18 | 0.25 | -8.91 | 33.12 | 4.29 |
| 3 | Constant | 18.35 | 9.10 | 2.02 | 0.05 | -0.32 | 37.02 | 19.90 |
|  | Kurtosis of Tilt Angle | -2.39 | 1.96 | -1.22 | 0.23 | -6.42 | 1.64 | 1.24 |
|  | Variance of Tilt Angle | 0.01 | 0.01 | 0.86 | 0.40 | -0.01 | 0.02 | 1.34 |
|  | Ratio of Acceleration | 21.88 | 23.39 | 0.94 | 0.36 | -26.11 | 69.86 | 2.35 |
|  | Mean of Acceleration | -9.47 | 11.71 | -0.81 | 0.43 | -33.50 | 14.57 | 3.09 |
|  | Sample Entropy (Angular Velocity) | 1.66 | 2.04 | 0.82 | 0.42 | -2.53 | 5.85 | 2.15 |
|  | Sample Entropy (Tilt) | 9.91 | 6.90 | 1.44 | 0.16 | -4.24 | 24.06 | 2.02 |
| 4 | Constant | 16.96 | 8.88 | 1.91 | 0.07 | -1.23 | 35.16 | 19.19 |
|  | Kurtosis of Tilt Angle | -2.43 | 1.95 | -1.24 | 0.22 | -6.42 | 1.57 | 1.24 |
|  | Variance of Tilt Angle | 0.01 | 0.01 | 0.85 | 0.41 | -0.01 | 0.02 | 1.34 |
|  | Ratio of Acceleration | 11.71 | 19.59 | 0.60 | 0.56 | -28.42 | 51.84 | 1.67 |
|  | Sample Entropy (Angular Velocity) | 0.92 | 1.81 | 0.51 | 0.62 | -2.79 | 4.63 | 1.72 |
|  | Sample Entropy (Tilt) | 9.11 | 6.78 | 1.34 | 0.19 | -4.79 | 23.00 | 1.98 |
| 5 | Constant | 17.79 | 8.62 | 2.06 | 0.05 | 0.16 | 35.42 | 18.55 |
|  | Kurtosis of Tilt Angle | -2.51 | 1.92 | -1.31 | 0.20 | -6.44 | 1.41 | 1.23 |
|  | Variance of Tilt Angle | 0.01 | 0.01 | 0.84 | 0.41 | -0.01 | 0.02 | 1.33 |
|  | Ratio of Acceleration | 13.74 | 18.93 | 0.73 | 0.47 | -24.97 | 52.46 | 1.60 |
|  | Sample Entropy (Tilt) | 10.70 | 5.94 | 1.80 | 0.08 | -1.46 | 22.85 | 1.56 |
| 6 | Constant | 18.03 | 8.55 | 2.11 | 0.04 | 0.58 | 35.48 | 18.52 |
|  | Kurtosis of Tilt Angle | -2.55 | 1.90 | -1.34 | 0.19 | -6.43 | 1.33 | 1.23 |
|  | Variance of Tilt Angle | 0.01 | 0.01 | 1.00 | 0.33 | -0.01 | 0.02 | 1.29 |
|  | Sample Entropy (Tilt) | 13.04 | 4.95 | 2.63 | 0.01 | 2.92 | 23.15 | 1.10 |
| 7 | Constant | 21.89 | 7.62 | 2.87 | 0.01 | 6.34 | 37.44 | 14.74 |
|  | Kurtosis of Tilt Angle | -3.30 | 1.75 | -1.88 | 0.07 | -6.86 | 0.27 | 1.04 |
|  | Sample Entropy (Tilt) | 14.17 | 4.82 | 2.94 | 0.01 | 4.33 | 24.00 | 1.04 |
| 8 | Constant | 18.58 | 7.71 | 2.41 | 0.02 | 2.88 | 34.29 | 13.96 |
|  | Sample Entropy (Tilt) | 16.03 | 4.91 | 3.27 | 0.00 | 6.04 | 26.02 | 1.00 |

Appendix 5 – Correlation between UEFM score and various sensor variables


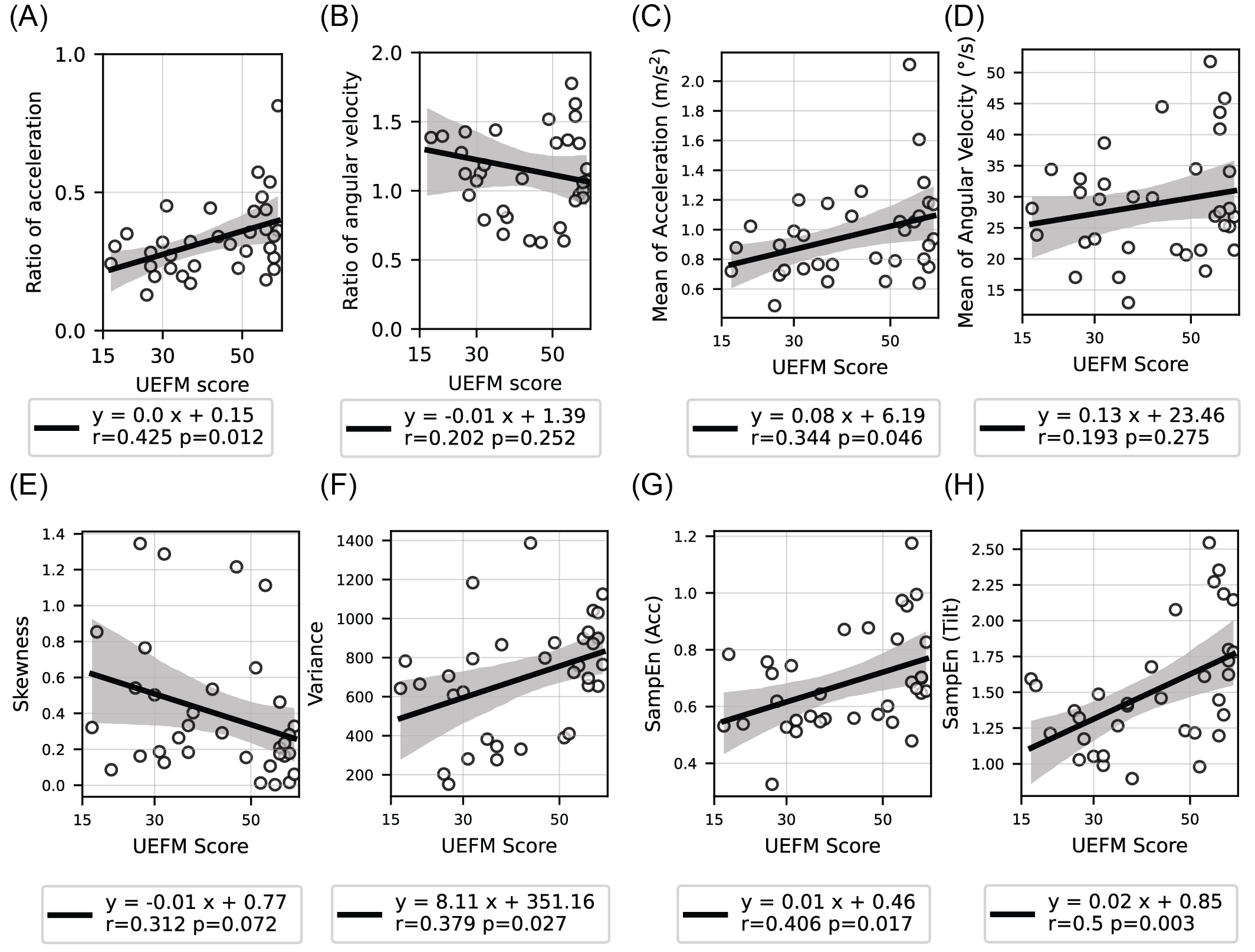


Figure 6. Correlation between UEFM score and various sensor variables. The shaded regions represent the 95% confidence interval. The correlation between the UEFM score and: (A) the ratio of acceleration in [1-3] m/s^2^ relative to [0-1] m/s^2,^ (B) The ratio of angular velocity in [5-15] deg/s relative to [0-5] deg/s. (C) the mean of acceleration, (D) the mean of angular velocity and the UEFM score, (E) skewness of tilt angle, (F) variance of tilt angle, (G) SampEn of acceleration (H) SampEn of angular velocity.

Appendix 6 – Model comparison throught the AIC and Correlations between features


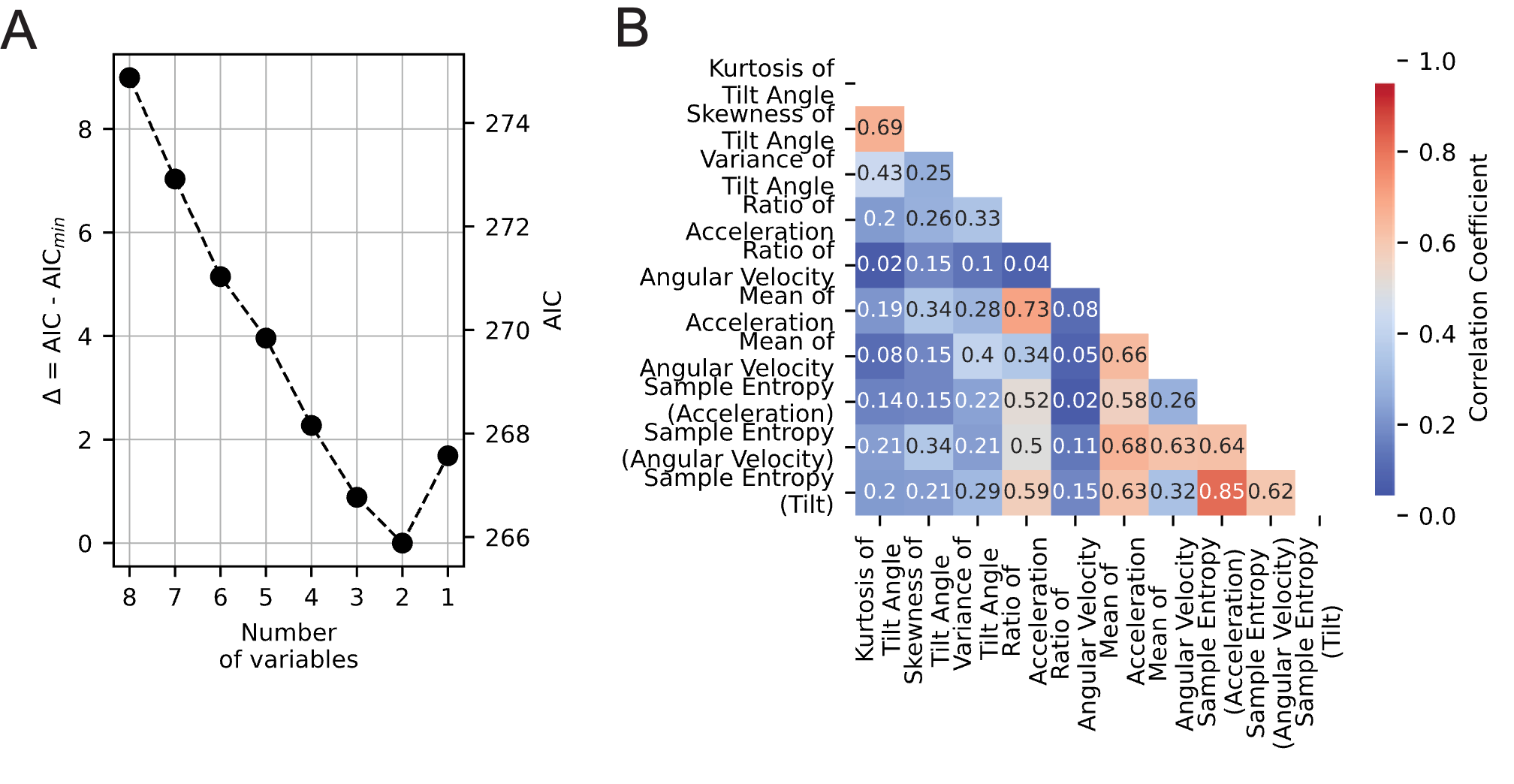


Figure 7. The variable selection analysis. (A) Model comparison through the AIC. (B) Correlations between features.
